# Supplementary material for: Breast Cancer Screening and Perceptions of Harm among Young Adults in Japan: Results of a Cross-Sectional Online Survey
Source: Curr Oncol. 2023 Feb 8;30(2):2073–87. doi: 10.3390/curroncol30020161 (PMC9955860; doi:10.3390/curroncol30020161)
Supplement: Supplementary file 1 [file curroncol-30-00161-s001.zip › curroncol-2148427-supplementary/Supplementary Material S1.docx]

Medical insurance and private insurance in this study cannot be merged. This section describes the health situation in Japan. The reasons for this are: ①Medical insurance is Japan's public medical insurance. Public health insurance is compulsory for all citizens. In other words, it is a system in which citizens support each other so that everyone can receive medical care with peace of mind. Private insurance, on the other hand, provides hospitalization and surgical benefits as the main policy, although the coverage varies by the insurance company. The major distinction between the two insurance policies is shown in Table 1. ②The questions for medical insurance and private insurance are different and cannot be merged. It is preferable to describe it as a characteristic of the health care system in the text. However, it would be redundant and should be explained only in the peer review reply.

Table 1. Distinction between medical insurance and private insurance

|  | Medical Insurance | Private Insurance |
| --- | --- | --- |
| Eligibility | Mandatory Enrollment | Voluntary Enrollment |
| Purpose of Membership | Stability of people's lives | Co-pay coverage |
| Insurance Premium | Depends on income | Varies by age, gender, and coverage |
| Benefit | Benefit in Kind | Receipt of insurance proceeds |
| Types and Systems | Health insurance, high-cost medical care, etc. | Medical insurance, cancer insurance, etc. |
