# Supplementary material for: Breast Cancer Screening and Perceptions of Harm among Young Adults in Japan: Results of a Cross-Sectional Online Survey
Source: Curr Oncol. 2023 Feb 8;30(2):2073–87. doi: 10.3390/curroncol30020161 (PMC9955860; doi:10.3390/curroncol30020161)
Supplement: Supplementary file 1 [file curroncol-30-00161-s001.zip › curroncol-2148427-supplementary/Supplementary Table S1.docx]

Table S1. All the personal characteristics used in this study.

| **Characteristic** | **Total  (n=1071)** |
| --- | --- |
| **Region** |  |
| Hokkaido | 42 (3.9%) |
| Tohoku | 75 (7.0%) |
| Kanto・Koshinetsu | 413 (38.6%) |
| Tokai・Hokuriku | 155 (14.5%) |
| Kinki | 168 (15.7%) |
| Chugoku・Shikoku | 86 (8.0%) |
| Kyushu・Okinawa | 132 (12.3%) |
| **Population size** |  |
| 23 wards of Tokyo or 1,000,000-city | 263 (24.6%) |
| 800,00-city | 105 (9.8%) |
| 300,000-city | 228 (21.3%) |
| 100,000-city | 234 (21.9%) |
| 50,00-city | 130 (12.1%) |
| Towns and villages | 111 (10.4%) |
| **Age** |  |
| 20-24 | 108 (10.1%) |
| 25-29 | 340 (31.8%) |
| 30-34 | 304 (28.4%) |
| 35-39 | 319 (29.8%) |
| **BMI^*^** |  |
| Lean | 226 (21.1%) |
| Normal | 743 (69.4%) |
| Obese | 102 (9.5%) |
| **Marital Status** |  |
| Married | 630 (58.8%) |
| Single | 441 (41.2%) |
| **Children** |  |
| Yes | 468 (43.7%) |
| No | 603 (56.3%) |
| **Household composition** |  |
| Single | 182 (17.0%) |
| 2-person household | 226 (21.1%) |
| 2-generation family | 559 (52.2%) |
| 3-generation family | 71 (6.6%) |
| Others | 33 (3.1%) |
| **Work status** |  |
| Self-employed | 35 (3.3%) |
| Regular employment | 478 (44.6%) |
| Parttime job | 190 (17.7%) |
| Student | 49 (4.6%) |
| Housewife | 268 (25.0%) |
| Unemployed | 51 (4.8%) |
| **Educational Background** |  |
| Primary and secondary school | 27 (2.5%) |
| High School | 236 (22.0%) |
| Junior colleges and vocational schools | 213 (19.9%) |
| University | 430 (40.2%) |
| Graduate School | 33 (3.1%) |
| Others | 132 (12.3%) |
| **Household income^**^** |  |
| No income | 18 (1.7%) |
| Less than $7,000 | 27 (2.5%) |
| $7,000-$22,000 | 115 (10.7%) |
| $22,000-$37,000 | 320 (29.9%) |
| $37,000-$51,000 | 284 (26.5%) |
| $51,000-$73,000 | 189 (17.7%) |
| $73,000-$110,000 | 91 (8.5%) |
| More than $110,000 | 27 (2.5%) |
| **Medical insurance^***^** |  |
| Association health insurance | 409 (38.2%) |
| Union health insurance | 171 (16.0%) |
| Mutual aid association | 104 (9.7%) |
| National health insurance | 282 (26.3%) |
| National health insurance association | 63 (5.9%) |
| Others | 16 (1.5%) |
| Unknown | 26 (2.4%) |
| **Medical insurance (dependent)** |  |
| Myself | 638 (59.6%) |
| Family | 433 (40.4%) |
| **Family history of breast cancer** |  |
| Yes | 96 (9.0%) |
| No | 975 (91.0%) |
| **Family history of cervical cancer** |  |
| Yes | 51 (4.8%) |
| No | 1020 (95.2%) |
| **Medical consultation** |  |
| Yes | 286 (26.7%) |
| No | 785 (73.3%) |
| **How is your current health condition** |  |
| Not good | 22 (2.1%) |
| Not so good | 159 (14.9%) |
| Usually | 567 (52.9%) |
| Well good | 202 (18.9%) |
| Good | 121 (11.3%) |
| **Are you taking care of your own health** |  |
| Not careful at all | 26 (2.4%) |
| Not very careful | 163 (15.2%) |
| Cannot say either way | 240 (22.4%) |
| Sometimes very carefully | 491 (45.9%) |
| Always be careful | 151 (14.1%) |
| **Do you have health concerns** |  |
| Very anxious | 93 (8.7%) |
| A little uneasy | 503 (47.0%) |
| Cannot say either way | 313 (29.2%) |
| No anxiety | 128 (12.0%) |
| No worries at all | 34 (3.2%) |
| **What to pay attention to for health** |  |
| Pay attention to diet |  |
| Yes | 665 (62.1%) |
| No | 406 (37.9%) |
| Get enough sleep |  |
| Yes | 677 (63.2%) |
| No | 394 (36.8%) |
| Don't drink too much alcohol |  |
| Yes | 394 (36.8%) |
| No | 677 (63.2%) |
| Not smoking cigarettes |  |
| Yes | 590 (55.1%) |
| No | 481 (44.9%) |
| Have regular health checkups |  |
| Yes | 246 (23.0%) |
| No | 825 (77.0%) |
| Do regular exercise |  |
| Yes | 243 (22.7%) |
| No | 828 (77.3%) |
| Avoid stress |  |
| Yes | 372 (34.7%) |
| No | 699 (65.3%) |
| Rinse your mouth and wash your hands |  |
| Yes | 507 (47.3%) |
| No | 564 (52.7%) |
| Other |  |
| Yes | 17 (1.6%) |
| No | 1054 (98.4%) |
| **The most feared disease** |  |
| Cancer | 459 (42.9%) |
| Heart disease | 65 (6.1%) |
| Brain Attack | 131 (12.2%) |
| Pneumonia | 6 (0.6%) |
| Diabetes | 72 (6.7%) |
| Liver disease | 14 (1.3%) |
| Dementia | 108 (10.1%) |
| Depression | 72 (6.7%) |
| Other | 20 (1.9%) |
| None | 124 (11.6%) |
| **Annual influenza vaccination status** |  |
| Vaccinated every year | 299 (27.9%) |
| Occasionally get vaccinated | 190 (17.7%) |
| Get vaccinated when I think about it | 67 (6.3%) |
| Don't get vaccinated | 391 (36.5%) |
| Don't need vaccinations | 124 (11.6%) |
| **Private medical insurance** |  |
| Yes | 557 (52.0%) |
| No | 514 (48.0%) |

^*^BMI: Lean (BMI<18.5), Normal(18.5≤BMI<25), and Obese (BMI≥25). ^**^Household income: Calculated according to the exchange rate on July 26, 2022 (1 yen = $0.0073). ^***^Employee insurance mainly includes "Association health insurance (for employees of small and medium-sized companies and their dependents)," "Union health insurance (for employees of large companies and their dependents)," "Mutual aid association (for public employees and their dependents)," and "National health insurance association (for doctors, construction workers and their dependents). Regional insurance includes "National health insurance (for people who are not covered by employee insurance, such as the self-employed and unemployed).
